# Biomimetic Brain-Targeted Delivery of Esterified XAV939 for Treating Autism-Associated Social Deficits

**DOI:** 10.3390/pharmaceutics18080951

**Published:** 2026-07-31

**Authors:** Hanze Liu, Ya-Rong Wang, Mengmeng Wang, Tiantian Yu, Daozhou Liu, Jing Wang, Yang Gao, Yuanyiyang Hu, Hongyu Ma, Feng Gao, Shengxi Wu, Zhao Wei, Yazhou Wang

**Affiliations:** 1 Yan’an Medical College, Yan’an University, Yan’an 716000, China; 2 Department of Neurobiology, School of Basic Medicine, Fourth Military Medical University, Xi’an 710032, China; 3 The Shaanxi Province Key Laboratory of Brain Function Analysis and Modulation, Xi’an 710032, China; 4 School of Life Sciences, Northwest University, No. 229, Taibai North Road, Xi’an 710069, China; 5 Department of Pharmaceutics, School of Pharmacy, Fourth Military Medical University, Xi’an 710032, China; 6 The Key Laboratory of Neural and Vascular Biology, Ministry of Education, Hebei Medical University, Shijiazhuang 050017, China; 7 Department of Neurobiology, Hebei Medical University, Shijiazhuang 050017, China; 8 Shaanxi Key Laboratory of Chiral Drug and Vaccine Adjuvants, School of Pharmacy, Fourth Military Medical University, Xi’an 710032, China

**Keywords:** autism spectrum disorder, social deficits, XAV939, biomimetic brain targeting

## Abstract

**Background/Objectives**: Autism spectrum disorder (ASD) is a group of developmental disorders featured by social dysfunction, for which there still lacks effective treatments. Our previous study demonstrated that XAV939, a tankyrase inhibitor, could alleviate social dysfunction in two ASD mouse models via suppressing Wnt and glycolytic signaling. However, its further application is limited by poor brain–blood barrier penetration and low bioavailability. **Methods**: XAV939 was structurally optimized by esterification. The effects of XAV939-derivatives on Wnt/glycolysis were assessed by Western blotting, Topflash luciferase assay, lactate levels and the extracellular acidification rate. Social behaviors were evaluated by a three-chamber test, a resident–intruder test and ultrasonic vocalization. Biomimetic brain targeting was achieved by neuron-astrocyte hybrid cell membrane encapsulation. Periphery toxicities were examined by biochemical and histological analysis. **Results**: Three esterified XAV939 were synthesized. Data from both 293FT cells and primary *Shank3b^-/-^* neurons revealed that an alkyl ester prodrug of XAV939 (named XAV939-L1) exhibited dual inhibition of Wnt/glycolysis. Intravenous injection of XAV939-L1 effectively improved the social function of *Shank3b^-/-^* mice but showed hepatic side effects. Further, we made brain-targeting XAV939-L1 (XAV939-L1-BT) by neuron–astrocyte hybrid cell membrane encapsulation, which greatly enhanced the accumulation of XAV939-L1-BT in the brain and reduced its distribution in peripheral tissues (liver and intestine). At a half-dose of XAV939-L1, XAV939-L1-BT exhibited significant social improvement effects without obvious hepatic and intestinal toxicity. **Conclusions**: Our data demonstrated an esterified XAV939 and its biomimetic brain-targeted formula as a potential drug candidate for treating ASD-associated social dysfunction.

## 1. Introduction

Autism spectrum disorder (ASD) is a neurodevelopmental condition characterized by core deficits in sociability, social interaction and communication [1]. As a lifelong disorder with no known cure, ASD imposes substantial economic burdens on families and society. In the United States, the estimated lifetime cost per individual with ASD reaches as high as $3.2 million [2]. Currently, the therapeutic approaches are largely concentrated on behavioral training and symptomatic management, with modest overall efficacy reported. The effective pharmacological interventions in clinical practice are mainly focused on the comorbid psychiatric symptoms. Robust translational evidence for core syndromes of ASD is still lacking [3]. Therefore, developing novel therapeutics for the social impairments will be helpful for improving the life quality of patients with ASD.

The etiology of ASD is complex, involving hundreds of risk genes and various environmental factors [4]. Although ASD is strongly associated with synaptic pathology [5], its etiological heterogeneity presents considerable challenges for drug development. Interventions targeting a single factor are likely to benefit only a subset of patients, underscoring the need to disclose the common molecular mechanisms underlying ASD. Recent transcriptomic analyses have identified dysregulation of the Wnt-β-catenin, PI3K–AKT and RAS–ERK signaling pathways as key aberrations associated with ASD [6]. Concurrently, metabolic abnormalities, reflected by an elevated lactate/pyruvate ratio, have been observed in approximately 30–80% of individuals with ASD [7,8], implicating that aberrant energy metabolism may offer a convergent point for intervention. Given the important roles of Wnt signaling in synaptic development [9,10], and the importance of a glucose-based energy supply for synaptic transmission [11,12], both Wnt signaling and glycolysis have been proposed as potential therapeutic targets, respectively [13,14,15]. Therefore, we speculate that simultaneously targeting Wnt–glycolysis signaling may offer a novel avenue for ASD drug discovery.

Our previous research demonstrated that canonical Wnt signaling and glycolysis are aberrantly upregulated in the neurons of two widely adopted ASD mouse models (*Shank3b^-/-^* and valproic acid-pretreated mice) [16,17]. A Wnt inhibitor, XAV939, could suppress glycolysis and improve the social function of these two ASD mouse models [16]. However, its therapeutic potential is limited by low bioavailability, systemic side effects, and poor brain delivery efficiency.

To overcome these limitations, we synthesized an esterified XAV939 derivative (named XAV939-L1) and prepared a biomimetic brain-targeted formulation of XAV939-L1 (XAV939-L1-BT), and demonstrated the efficacy of XAV939-L1-BT in treating the social deficiency in the ASD mouse model (*Shank3b^-/-^*) mice with minimized peripheral side effects.

## 2. Materials and Methods

### 2.1. Synthesis of XAV939 Derivatives and In Vitro Screening

Synthesis of XAV939-L1: 4-(Trifluoromethyl)phenylurea (0.39 g, 2 mmol) and methyl 4-oxo-3,4-dihydrothieno[3,4-d]pyrimidine-3-carboxylate (0.36 g, 2 mmol) were dissolved in methanol (10 mL). Anhydrous K_2_CO_3_ (0.55 g, 4 mmol) was added, and the mixture was stirred under reflux for 12 h. After cooling down, the mixture was filtered, and the resulting filtrate was concentrated in vacuo to approximately 1–2 mL. Water was added, and then, the pH was adjusted to 3–4 with 2 M HCl, leading to precipitation. The solid was collected by vacuum filtration and dried at 60 °C for 10 h to generate the product as a white solid (0.45 g, 71% yield).

Synthesis of XAV939-L2: XAV939 (0.30 g, 1 mmol) was dissolved in THF (10 mL). Triethylamine (0.21 g, 2 mmol) and acetyl chloride (0.10 g, 1.3 mmol in 2 mL THF) were added sequentially. The mixture was stirred at room temperature for 6 h. The product was purified by silica gel column chromatography (DCM/MeOH = 20:1, *v*/*v*) to yield a white solid (0.28 g, 80% yield).

Synthesis of XAV939-L3: Following the synthetic method used for XAV939-L2, XAV939 was reacted with dimethylcarbamoyl chloride to obtain the target compound as a white solid (0.12 g, 63% yield).

The structures were confirmed by ^1^H NMR, ^13^C NMR, and HRMS. NMR spectra were recorded on a Bruker-400 MHz spectrometer using CDCl_3_ or DMSO-d_6_ as solvents, with tetramethylsilane (TMS) as an internal standard. Chemical shifts (δ) are reported in ppm and coupling constants (J) in Hz. Column chromatography was performed on silica gel (200–300 mesh), and thin-layer chromatography (TLC) was monitored under a 254 nm UV lamp. High-resolution mass spectra (HRMS) were obtained on an Agilent 6550 iFunnel Q-TOF LC/MS system equipped with an electrospray ionization (ESI) source.

For in vitro screening, 293FT cells (catalog/accession no. BNCC353535, BeNa Culture Collection, Beijing Beina Chuanglian Biotech Institute, Beijing, China) were treated with 20 μM XAV939, XAV939L1, XAV939L2, and XAV939L3 for 3 days. Then, cells were collected for assessing Wnt signaling and glycolysis.

### 2.2. Animals

Wild-type mice (male C57BL/6 mice, E14-17 pregnant mice and P2 neonatal pups) were bought from the animal core facility of the Fourth Military Medical University. *Shank3b^-/-^* mice were a kind gift from Prof. Guoping Feng as described [18]. They were housed under a 12 h light/dark cycle at 23–25 °C with free access to water and food. All of the animal experiments were approved by the FMMU ethics committee for experimental animals (Approval code: 20260015, approval date: 26 March 2026; approval code: 20210122, approval data: 24 January 2021.) and conducted according to the ARRIVE guidelines to reduce any suffering or stress of the animals. The overall conditions of the animals, including the possible adverse effects, were recorded during treatment. The humane endpoint was complied with the guidelines of the experimental animal care and use committee of FMMU.

### 2.3. Western Blotting

Cells or tissues were lysed in pre-chilled RIPA buffer containing protease and phosphatase inhibitor cocktails on ice for 30 min. Lysates were centrifuged at 12,000× *g* for 10–15 min at 4 °C, and the supernatant was collected. Protein concentrations were determined by BCA assay kit. Protein samples were separated by 10–12% SDS-PAGE and transferred onto PVDF membranes. Membranes were blocked with 5% non-fat milk or BSA in TBST, followed by incubation with primary antibodies overnight at 4 °C. After washing, membranes were re-incubated with HRP-conjugated secondary antibodies for 1 h at room temperature. Protein bands were visualized using an enhanced chemiluminescence (ECL) kit (Thermo Fisher Scientific, Waltham, MA, USA) and imaged with a Tanon gel imaging system. Densitometric analysis was performed using Image J software (Version 2.14.0; National Institutes of Health, Bethesda, MD, USA). Relative protein levels were normalized to the GAPDH control and presented as fold change relative to the control group. The followings are the antibodies used: ① rabbit anti-Non-phospho (Active) β-Catenin (Ser45) antibody (1:1000, Cat# 19807, Cell Signaling Technology, Danvers, MA, USA); ② rabbit anti-Axin 2 antibody (1:100, Cat# ab107613, Abcam, Cambridge, UK); ③ rabbit anti-Phospho-β-Catenin (Ser33/37/Thr41) antibody (1:1000, Cat# 9561, Cell Signaling Technology, Danvers, MA, USA); ④ rabbit anti-Phospho-GSK-3β (Ser9) antibody (1:1000, Cat# 5558, Cell Signaling Technology, Danvers, MA, USA); ⑤ mouse anti-GAPDH antibody (1:50,000–1:500,000, Cat# 60004-1-Ig, Proteintech, Wuhan, China); ⑥ goat anti-mouse IgG (H + L), HRP conjugate (1:50,000, Cat# G21040, RRID: AB_2536527, Invitrogen, Carlsbad, CA, USA); and ⑦ goat anti-rabbit IgG (H + L), HRP conjugate (1:50,000, Cat# G21234, RRID: AB_2536530, Invitrogen).

### 2.4. Lactate Measurement

Lactic acid levels were measured using an L-Lactic Acid Assay Kit (WST method, Cat# BC5345, Solarbio, Beijing, China). For tissues, the samples were homogenized in ice-cold Extraction Solution I (1:5–10, *w*/*v*), centrifuged at 12,000× *g* for 10 min at 4 °C, and the supernatant was collected and treated with Extraction Solution II, followed by another centrifugation. For cells, 5 × 10^6^ cells were homogenized in 1 mL Extraction Solution I by ultrasonication on ice and centrifuged as described above. For the assay, samples or standards and working solution were added to a 96-well plate and incubated at 37 °C for 30 min in the dark. The reaction was stopped by adding distilled water, and absorbance was measured at 450 nm. Lactate concentrations were calculated based on a standard curve.

### 2.5. Biochemical Analysis of Hepatic Function

Mouse blood samples were collected via orbital bleeding into tubes without anticoagulant and allowed to clot for 30 min. Serum was separated by centrifugation at 3000 rpm for 10 min at 4 °C and stored at −80 °C. The activities of alkaline phosphatase (ALP), alanine aminotransferase (ALT), aspartate aminotransferase (AST), and total bile acid (TBA) levels in serum were measured using an automated biochemical analyzer at Wuhan Servicebio Technology Co., Ltd. (Wuhan, China) .

### 2.6. Extracellular Acidification Rate (ECAR)

ECAR was measured using a Seahorse XF-24 Extracellular Flux Analyzer as described [19]. Cells were seeded in Seahorse cell culture plates at a density of 8 × 10^4^ cells per well. The sensor cartridge was hydrated with calibrant overnight at 37 °C in a non-CO_2_ incubator. During the assay, glucose (10 mM), oligomycin (1 μM), and 2-deoxyglucose (50 mM) were injected sequentially, and ECAR was measured at each time point. Each measurement cycle consisted of 2 min mix, 1 min wait, and 3 min measure. After the assay, the cells were counted, and ECAR values were normalized to cell number (pmol/min/10^4^ cells).

### 2.7. Behavioral Tests

Three-chamber social test: The apparatus was a rectangular, opaque acrylic box divided into three chambers. The test consisted of two phases. In the sociability phase, the subject mouse was habituated in the center chamber and then allowed to freely explore the three chambers, with one side containing a stranger mouse in a cylindrical cage and the other side empty. The time spent in each side chamber was recorded. In the social novelty phase, a second stranger mouse was placed in the previously empty side cage, and the time spent exploring the familiar (first stranger) vs. the novel (second stranger) mouse was recorded. Behavioral trajectories were recorded and analyzed using SMART 3.0 software. The social preference index was calculated as (time in social side—time in non-social side)/(time in social side + time in non-social side).

Resident-intruder test: After 24 h of single housing, a smaller, same-strain, same-sex intruder mouse was put into the resident cage of the test mouse. Interactions were video recorded for 10 min. The duration and frequency of resident-initiated social contacts (e.g., sniffing, following, grooming) towards the intruder were analyzed.

Ultrasonic vocalization (USV) recording: Subject mice were placed in a cage inside a sound-attenuating chamber, and a same-strain female mouse was introduced to elicit USVs. Vocalizations were recorded for 10 min using an ultrasonic microphone (20–120 kHz range) at a 250 kHz sampling rate. Data were acquired using SeaWave 2.0 software and analyzed using the Deep Squeak 3.0 toolkit (MATLAB R2018a platform) to quantify the number, average duration, and peak frequency of calls in the 20–120 kHz range.

Open field test: The open field test was adopted to evaluate spontaneous locomotor activity and anxiety-like behavior. Before testing, mice were acclimated to the behavioral testing room. Each mouse was then placed individually in the center of a 50 × 50 × 50 cm open-field arena and allowed to explore freely for 10 min. Locomotor activity was recorded. The percentage of time spent in the center zone, the percentage of distance traveled in the center zone, and total distance traveled were quantified for subsequent analysis.

Hanging wire test: The hanging wire test was used to assess the neuromuscular function. Briefly, each mouse was gently placed at the center of a horizontal metal wire (approximately 35 cm above a soft bedding layer) and allowed to grasp the wire with its forelimbs. The latency of the mouse falling from the wire was recorded as the hanging time. A maximum cutoff time was set as 600 s. Mice that fell before reaching the cutoff time were allowed up to two additional trials, with a 1 min rest interval between trials. The longest hanging time obtained from the trials was used for subsequent statistical analysis.

Rotarod test: The rotarod test was adopted to assess motor coordination and balance. Mice were trained for three consecutive days before the formal test. On days 1 and 2, mice were trained on the rotarod at constant speeds of 5 rpm and 10 rpm, respectively, for 3 min per trial. On day 3, mice were trained under an accelerating protocol from 4 to 20 rpm over 300 s. On day 4, mice were tested with the rod accelerating from 4 to 40 rpm within 90 s, followed by a constant speed of 40 rpm for 210 s. Each mouse was subjected to 3 trials per day with an inter-trial interval of at least 20 min. The latency to fall was recorded manually, with a maximum cutoff time of 300 s. The average latency of three trials on the test day was used for analysis.

### 2.8. H.E. Staining

Mice were sacrificed and perfusion fixed with 4% PFA. Then, duodenum, jejunum, and ileum tissues were collected. Tissues were immediately post-fixed in 10% neutral buffered formalin for 24 h. Subsequently, they were dehydrated in graded ethanol and embedded in paraffin. Sections were stained with hematoxylin and eosin (H&E). The intestinal morphology, including villi integrity, glandular arrangement and mucosal thickness, was examined using a light microscope.

### 2.9. Primary Neural Culture

Primary cortical neurons and astrocytes were typically obtained from E14-17 mouse embryos or P2 neonatal pups, respectively. Under sterile conditions, the cerebral cortex was dissected, and the covering meninges and blood vessels were carefully removed. After tissue digestion, a single-cell suspension was prepared by mechanical trituration. For neuronal culture, cells were seeded onto poly-L-lysine-coated dishes and cultured in Neurobasal medium supplemented with B27. For astrocyte culture, cells were seeded in DMEM containing 10% fetal bovine serum. After reaching confluence, astrocytes were purified by shaking at 260 rpm overnight at 37 °C.

### 2.10. Cell Membrane Extraction

Cell membranes were isolated by differential centrifugation. Adherent neurons or astrocytes were collected, digested with 0.25% trypsin, and pelleted by centrifugation. The cell pellet was resuspended in ice-cold lysis buffer (10 mM KCl, 2 mM MgCl_2_, 20 mM Tris-HCl, pH 7.5, with protease inhibitors) and lysed on ice for 15 min. The lysate was subjected to sequential centrifugation: 700× *g* for 10 min to remove nuclei and unbroken cells, 12,000× *g* for 10 min to pellet mitochondria and other organelles, and finally 140,000× *g* for 45 min to pellet the membrane fraction. The membrane pellet was resuspended in sterile nuclease-free water, lyophilized, and stored at −80 °C.

### 2.11. Preparation of Biomimetic Brain-Targeted XAV939-L1 (XAV939-L1-BT) and Morphological Characterization

Soybean phosphatidylcholine (SPC, 90 mg), cholesterol (4 mg), DSPE-PEG2000 (9 mg), Cy5.5 (4 mg), and XAV939-L1(8 mg) were dissolved in chloroform (2 mL). The mixture was transferred, and the organic solvent was removed, forming a thin lipid film on the flask wall. The film was hydrated with an appropriate volume of ultrapure water to obtain a crude liposome suspension. The suspension was sequentially sonicated and extruded through 100 nm polycarbonate membranes using a liposome extruder to achieve uniform size. Pre-prepared astrocyte and neuronal cell membrane powders (50 mg each) were mixed with the liposome suspension, sonicated in an ice-water bath for 30 s, and incubated at room temperature for 2 h to allow spontaneous coating/fusion of membrane fragments onto the liposome surface, yielding the final biomimetic liposomes. The product was aliquoted, mixed with lyoprotectants, lyophilized, and stored at −20 °C for use.

The above-described nanoparticle morphology was observed using a transmission electron microscope (TEM). Briefly, samples were diluted to an appropriate concentration with ultrapure water, dispersed uniformly by ultrasonication, and dropped onto carbon-coated copper grids. The grids were air-dried, mounted onto the sample holder, and inserted into the TEM instrument. After locating the target area at low magnification, morphology was observed, and images were captured at high magnification.

### 2.12. Particle Size and Zeta Potential Characterization of XAV939-L1-BT

Particle size distribution and zeta potential were determined using a Brookhaven 90Plus particle size analyzer equipped with a 640 nm He-Ne laser. A 10 μL sample aliquot was diluted to 2 mL with ultrapure water, dispersed by ultrasonication, and transferred to a sample well. The mean hydrodynamic diameter and polydispersity index (PDI) were measured by dynamic light scattering DLS. Subsequently, an electrode was inserted into the same sample, and the instrument was switched to phase analysis light scattering mode to measure electrophoretic mobility via laser Doppler velocimetry, from which the zeta potential was calculated. All measurements were performed at 25 °C.

### 2.13. Fluorescence Spectroscopy of XAV939-L1-BT

Fluorescence spectra of the above nanoparticle of XAV939-L1-BT were performed using a Tecan multifunctional microplate reader. Briefly, a 10 μL sample aliquot was diluted to 2 mL with ultrapure water, dispersed uniformly by ultrasonication, and transferred to a black 96-well plate. Emission spectra were scanned across a full wavelength range using an appropriate excitation wavelength. The maximum emission wavelength and corresponding fluorescence intensity were recorded at 704 nm. To check the fusion of neuron–astrocyte membrane with XAV939 exosomes, neuronal membrane and astrocytic membrane were labeled with Cy3.3 and FITC, respectively. XAV939-L1-BT was examined by Leica STELLARIS STED.

### 2.14. In Vivo Fluorescence Imaging of XAV939-L1-BT

XAV939-L1-BT (2.5 mg/kg) was administered intravenously via the tail vein at a volume calculated based on body weight. At 12 h post-administration, mice were sacrificed under light-protected conditions. Brain, liver, kidney, duodenum, jejunum, and ileum tissues were dissected, respectively. Fluorescence images of the whole-mount organs were acquired using an IVIS Spectrum imaging system under identical exposure settings. Images were analyzed with Living Image software (version 4.8.0; PerkinElmer, Waltham, MA, USA). The mean fluorescence intensity was measured in regions of interest of the same size for each organ, and the background signal was subtracted.

### 2.15. High Performance Liquid Chromatography (HPLC)

Brain tissue homogenate was made by mechanical homogenization of 100 mg brain tissue in 400 μL saline and subsequent centrifugation at 12,000 rpm for 10 min. XAV939-L1 (100 μg/mL) was incubated with the brain homogenate for different time points. Methanol was used to stop the reaction, and HPLC was then conducted. Liquid chromatographic analysis was performed using an Agilent 1290 Infinity high-performance liquid chromatography system equipped with a diode array detector (DAD). After sample pretreatment, chromatographic separation was achieved on a Phenomenex C18 column (100 mm × 2.1 mm, 2.6 μm) maintained at 40 °C. The mobile phase consisted of 0.1% formic acid in water as solvent A and acetonitrile as solvent B. Gradient elution was performed at a flow rate of 0.40 mL/min with an injection volume of 2 μL. The gradient program was as follows: 0–1.0 min, 90% A and 10% B; 1.0–4.0 min, 10% B to 90% B; 4.0–8.0 min, 10% A and 90% B; 8.0–8.1 min, 90% B to 10% B; and 8.1–10.0 min, 90% A and 10% B for column re-equilibration. The detection wavelengths were set at 254 nm and 292 nm. Data acquisition, peak integration, and quantitative analysis were performed using Agilent OpenLab software (version LTS 01.11 [259], Santa Clara, CA, USA).

### 2.16. Statistical Analysis

Data collection was performed according to the rules of randomization. All data points were included for analysis, and the analyses were conducted by investigators blinded to the experimental design. Two-tailed Student’s *t*-test and one-way ANOVA followed by Tukey’s post hoc test were conducted. Prism 9.0 software (GraphPad Software Inc., La Jolla, CA, USA) was adopted for analysis. *p* < 0.05 was considered statistically significant. Data are presented as the mean ± standard error of the mean (S.E.M).

## 3. Results

### 3.1. Synthesis of XAV939 Derivatives via Esterification Modification

**Figure 1:**
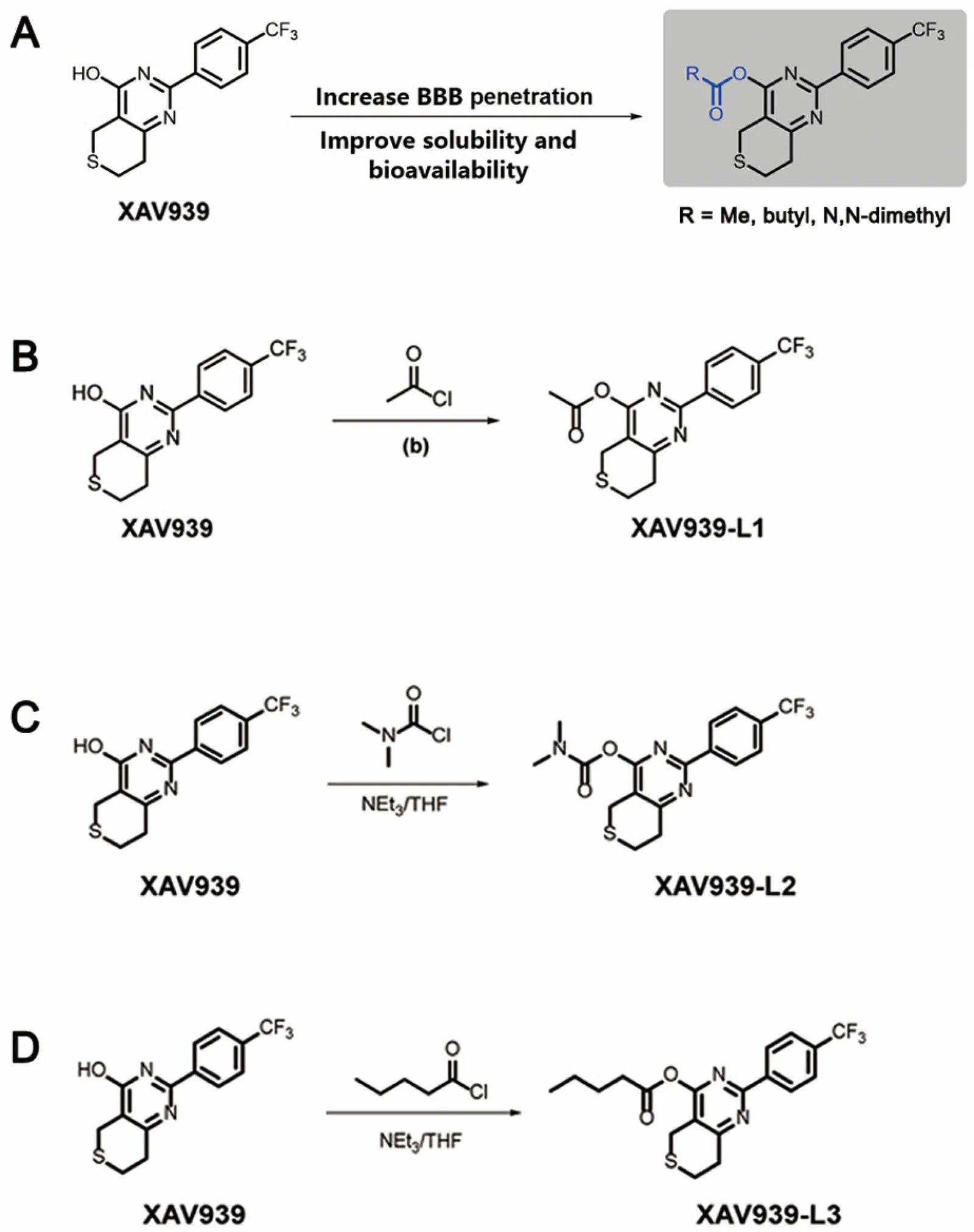
Synthesis of esterified XAV939. (**A**) Strategy of XAV939 modification. (**B**) Synthesis of XAV939-L1. (**C**) Synthesis of XAV939-L2. (**D**) Synthesis of XAV939-L3.

We synthesized three lipophilic prodrugs of XAV939 through esterification modification (Figure 1A). The target product of XAV939-L1 was obtained via reaction of XAV939 with an acetyl chloride under organic base conditions (Figure 1B). The compound XAV939-L2 was synthesized following a procedure analogous to that of XAV939-1, using XAV939 and dimethylcarbamoyl chloride as the starting materials (Figure 1C). The compound XAV939-L3 was synthesized using XAV939 and pentanoyl chloride as the starting materials (Figure 1D). The structures of these compounds were verified by ^1^H and ^13^C NMR and HRMS spectra (Supplementary Figure S1).

### 3.2. Identification of XAV939-L1 as a Dual Inhibitor of Wnt/Glycolysis Signaling

**Figure 2:**
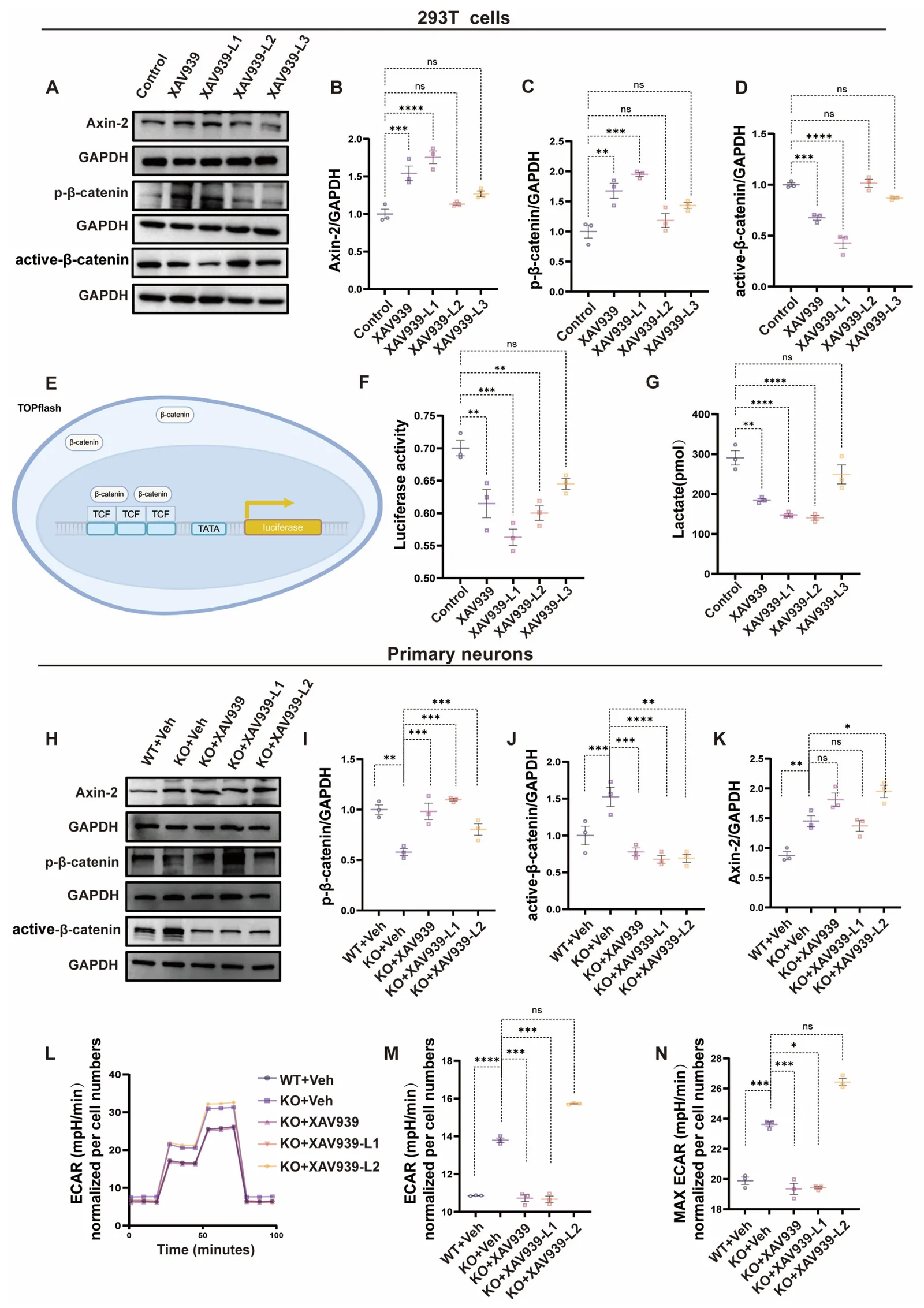
In vitro screening of potent Wnt/glycolysis dual inhibitors. (**A**–**D**) Western blotting and quantification of Axin2, p-β-catenin and active β-catenin in 293T cells treated with vehicle, XAV939, XAV939-L1, XAV939-L2 and XAV939-L3. (**E**,**F**) Topflash activity in 293T cells treated with vehicle, XAV939, XAV939-L1, XAV939-L2 and XAV939-L3. E, Created in BioRender. Liu, H. (2026) https://BioRender.com/cq80q1f (**G**) Lactate levels in 293T cells treated with vehicle, XAV939, XAV939-L1, XAV939-L2 and XAV939-L3. (**H**–**K**) Western blotting and quantification of Axin2, p-β-catenin and active β-catenin in primary neurons treated with vehicle, XAV939, XAV939-L1 and XAV939-L2. (**L**–**N**) ECAR assay of primary neurons treated with vehicle, XAV939, XAV939-L1 and XAV939-L2. Notice that only XAV939-L1 showed dual Wnt/glycolysis inhibition in both 293T cells and primary *Shank3b^-/-^* neurons. Data are presented as means ± S.E.M. One-way ANOVA followed by Tukey’s post hoc test. *n* = 3 batches of cells. * *p* < 0.05, ** *p* < 0.01, *** *p* < 0.001, **** *p* < 0.0001. WT, wild type. KO, *Shank3b^-/-^*. ns, non-significance.

The dual inhibitory effects of the aforementioned compounds on Wnt/glycolysis signaling were initially evaluated in 293T cells, using the same dose of XAV939 (20 μM) as a positive control. Wnt signaling was evaluated through the protein levels of Axin2 (a feedback target gene of Wnt signaling), active β-catenin (non-phosphorylated β-catenin at Ser45, which enters the nucleus to activate Wnt signaling), and phosphorylated β-catenin (Ser33/37/Thr41, p-β-catenin), which mediates the degradation of β-catenin, as well as Topflash luciferase activity, which reflects the transcriptional activity of nuclear β-catenin/TCF complex. Similar to XAV939, XAV939-L1 markedly increased the protein levels of Axin2 and phosphorylated β-catenin, while decreasing the protein levels of active β-catenin (Figure 2A–D). In the Topflash assay, both XAV939-L1 and XAV939-L2 significantly suppressed the levels of luciferase activity (Figure 2E,F). To assess glycolytic activity, we measured intracellular lactate concentrations in cells subjected to the same treatment. In addition to XAV939, both XAV939-L1 and XAV939-L2 significantly reduced the lactate levels (Figure 2G). These data suggest that XAV939-L1 and XAV939-L2 can inhibit Wnt/glycolysis signaling in 293T cells with an efficacy comparable to that of XAV939.

Next, we sought to identify the compound with more consistent dual inhibition using primary cortical neurons derived from *Shank3b^-/-^* mice. Regarding Wnt signaling-related proteins, both XAV939-L1 and XAV939-L2 significantly increased p-β-catenin levels and decreased active β-catenin levels in *Shank3b^-/-^* neurons, as compared with vehicle control (Figure 2H–J). XAV939-L2 also significantly elevated the levels of Axin2 (Figure 2H,K). To precisely assess the glycolytic status in primary neurons, we measured the extracellular acidification rate (ECAR), which reflects the proton efflux resulting from glucose conversion to lactate. The results showed that only XAV939-L1 significantly suppressed both the average and maximum ECAR in *Shank3b^-/-^* neurons, as XAV939 did (Figure 2L–N). These findings suggest that XAV939-L1 is reliable in inhibiting Wnt/glycolysis signaling in ASD neurons.

### 3.3. Amelioration of ASD Social Deficits via Intravenous Administration of XAV939-L1 with Hepatic and Intestinal Side Effects

To test if XAV939-L1 could be degraded into XAV939 in the brain, we incubated XAV939-L1 with brain tissue homogenate and measured the changes in XAV939-L1 and XAV939 at different time points. High-performance liquid chromatography showed that XAV939-L1 decreased by approximately 60% and XAV939 increased dramatically from 1 h of incubation, illustrating the rapid conversion of XAV939-L1 into XAV939 (Supplementary Figure S2).

**Figure 3:**
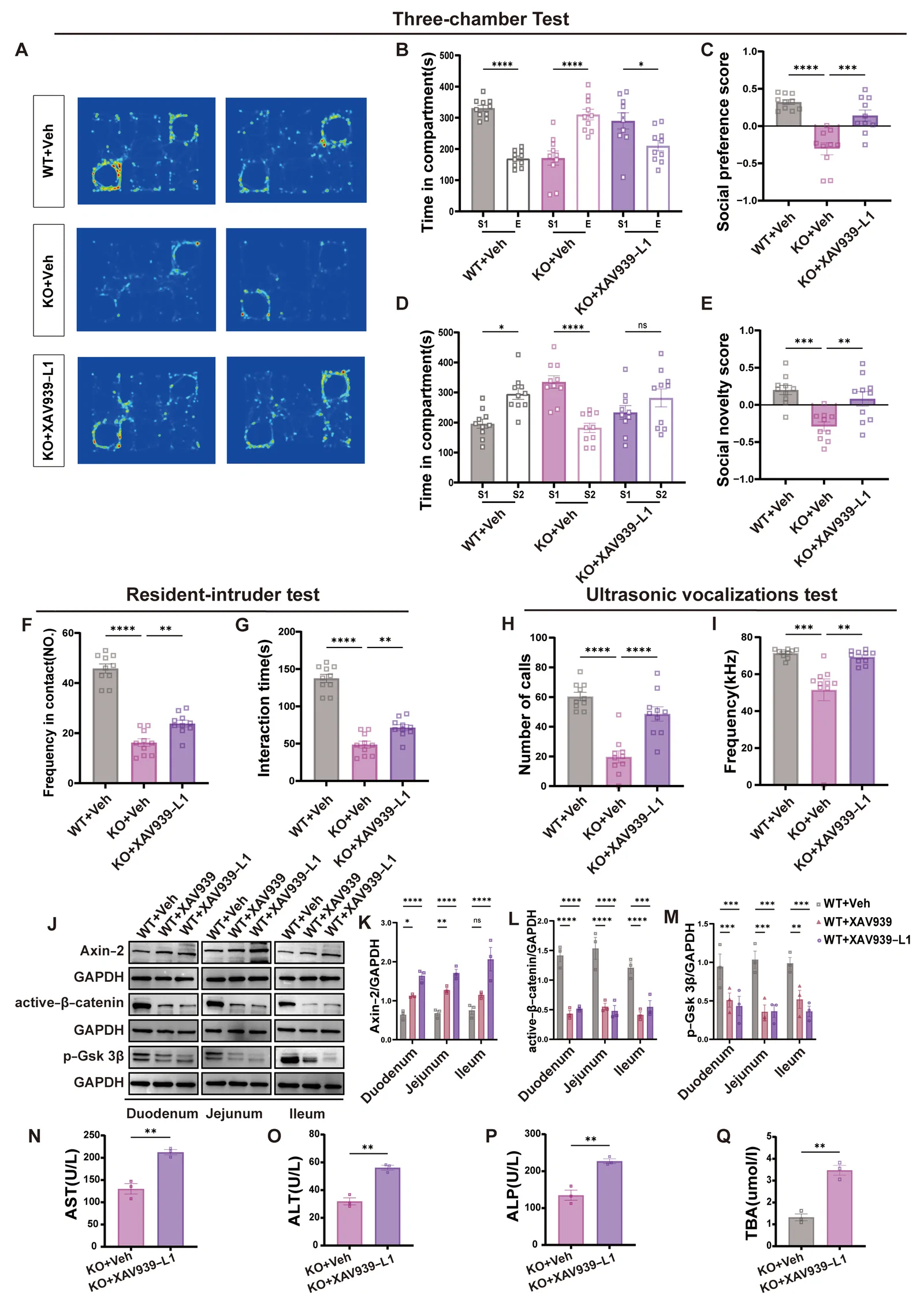
The social-improving effects and peripheral toxicity of XAV939-L1. (**A**–**E**) Three-chamber assay of WT mice treated with vehicle, *Shank3b^-/-^* mice treated with vehicle or XAV939-L1. (**F**,**G**) Resident-intruder assay of WT mice treated with vehicle, *Shank3b^-/-^* mice treated with vehicle or XAV939-L1. (**H**,**I**) Ultrasonic vocalization of WT mice treated with vehicle, *Shank3b^-/-^* mice treated with vehicle or XAV939-L1. Notice that intravenous injection of XAV939-L1 can attenuate the social dysfunction of *Shank3b^-/-^* mice. (**J**–**M**) Western blotting of Axin2, p-GSK-3β and active β-catenin in different segments of intestine. (**N**–**Q**) Hepatic function test of *Shank3b^-/-^* mice treated with vehicle or XAV939-L1 at the same dosage as the behavior study. Notice the Wnt inhibition in intestine and impairment of hepatic function by XAV939-L1. Data are presented as means ± S.E.M. One-way ANOVA followed by Tukey’s post hoc test. Two-tailed Student’s *t*-test. n = 10 mice per group in (**A**–**I**), 3–4 batches of cells in (J), 3 mice per group in (**N**–**Q**). * *p* < 0.05, ** *p* < 0.01, *** *p* < 0.001, **** *p* < 0.0001. WT, wild type. KO, *Shank3b^-/-^*. Veh, vehicle. ns, non-significance.

Since systemic administration of XAV939 shows very low bioavailability, we investigated whether intravenous administration of XAV939-L1 could ameliorate social deficits in *Shank3b^-/-^* mice. Six-week-old *Shank3b^-/-^* mice received daily intravenous injections of XAV939-L1 (5 mg/kg) for five consecutive days, and social behaviors were assessed 24 h after the final injection. In the three-chamber test, XAV939-L1 treatment significantly enhanced both social preference and social novelty in *Shank3b^-/-^* mice, compared to the vehicle treatment (Figure 3A–E). The resident-intruder test revealed that XAV939-L1 significantly increased the social interaction frequency and duration of *Shank3b^-/-^* mice with intruder mice (Figure 3F,G). Ultrasonic vocalization (USV), a key measure of social communication in mice, was impaired in *Shank3b^-/-^* mice. XAV939-L1 significantly increased both the number and frequency of USV calls emitted by *Shank3b^-/-^* male mice during the male–female interactions (Figure 3H,I). These data demonstrate that intravenous administration of XAV939-L1 can ameliorate social impairments in *Shank3b^-/-^* mice.

Considering that the liver bears high glycolytic activity [20], and the small intestine has high Wnt signaling under normal conditions [21], we next evaluated the potential peripheral toxicity of XAV939-L1 compared to XAV939 at an equivalent dosage, focusing on the small intestine and liver. In distinct intestinal segments (duodenum, jejunum, ileum), XAV939-L1 significantly increased Axin2 levels while decreasing active β-catenin and phosphorylated GSK-3β levels as XAV939 did (Figure 3J–M), indicating the inhibition of Wnt signaling in the small intestine by intravenous delivery of XAV939-L1. Blood biochemical analysis revealed that XAV939-L1 significantly elevated the activities of aspartate aminotransferase (AST), alkaline phosphatase (ALP), alanine aminotransferase (ALT), and the levels of total bile acid (TBA) in *Shank3b^-/-^* mice (Figure 3N–Q). These results indicate that, while intravenous XAV939-L1 effectively improves social function, it may induce potential peripheral side effects, such as hepatotoxicity and enterotoxicity.

We next assessed if the same XAV939-L1 treatment would affect the basal activity, locomotion, balance or grip strength of the mice. The open-field test, which assesses the animal’s basal movement, showed that, although *Shank3b^-/-^* mice exhibit somewhat reduced basal movement, XAV939-L1 did not affect the movement of both WT and *Shank3b^-/-^* mice (Supplementary Figure S3A–C). In the rotarod test, which reflects motor-sensory coordination, XAV939-L1 did not affect the latency time (Supplementary Figure S3D,E). In the hanging wire test, which assesses the griping capacity, XAV939-L1 did not alter the hanging time of both WT and *Shank3b^-/-^* mice (Supplementary Figure S3F,G). These data indicate that XAV939-L1, at the dosage used to improve social function, did not affect the animal’s basal movement.

### 3.4. Preparation and In Vivo Distribution of Brain-Targeted XAV939-L1 (XAV939-L1-BT)

**Figure 4:**
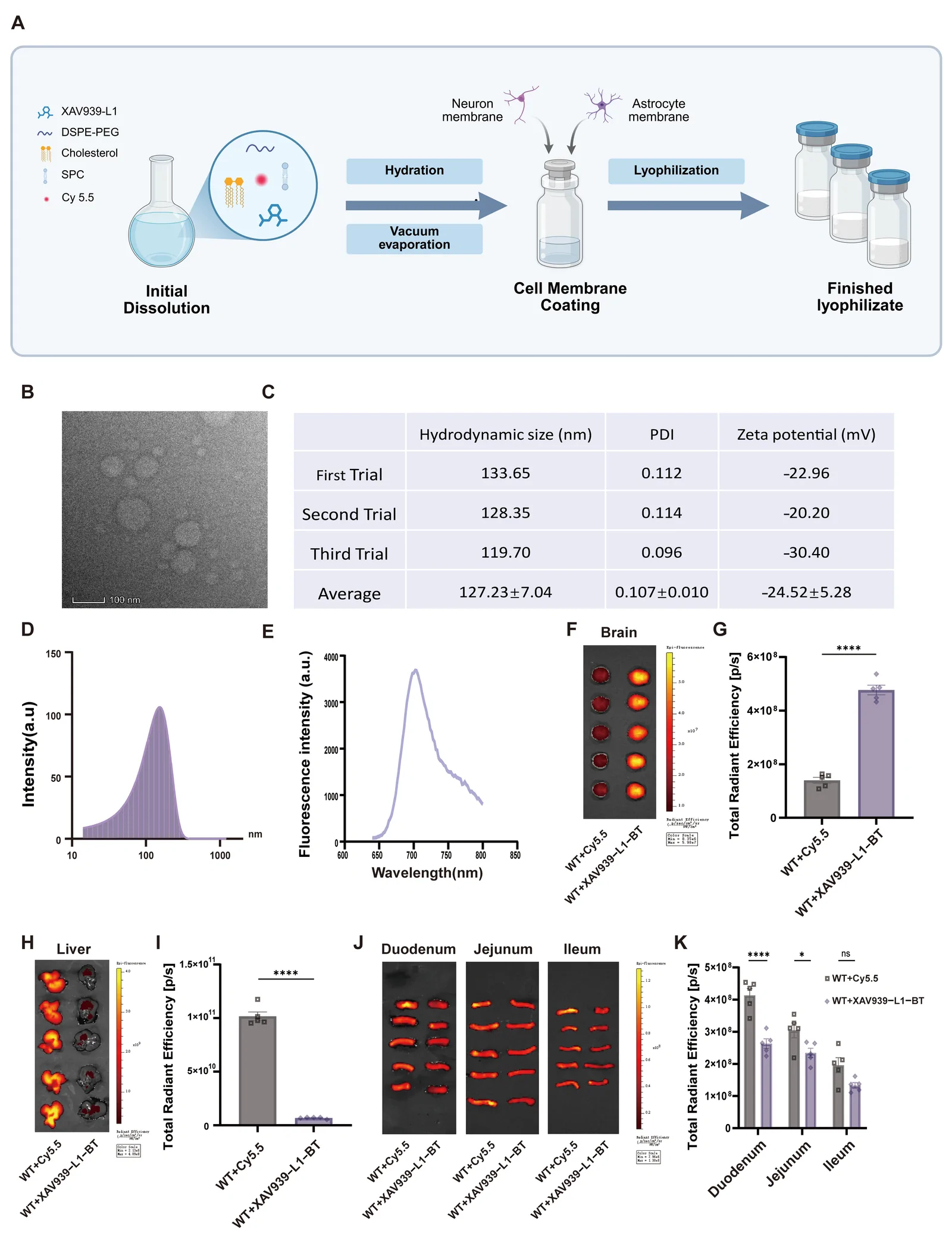
Physical properties and in vivo distribution of XAV939-BT. (**A**) Hybrid cell membrane coating of XAV939-L1 (XAV939-BT). Created in BioRender. Liu, H. (2026) https://BioRender.com/rebfbic. (**B**) Electron microscopic image of XAV939-BT nanoparticle. (**C**–**E**) Physical properties of XAV939-L1-BT. (**F**–**K**) Distribution of XAV939-BT in brain, liver and small intestine. Notice that XAV939-BT is highly enriched in brain. Data are presented as means ± S.E.M. * *p* < 0.05, **** *p* < 0.0001. One-way ANOVA followed by Tukey’s post hoc test. Two-tailed Student’s *t*-test. n = 5 mice per group in (**G**,**I**,**K**). WT, wild type. ns, non-significance.

To increase the therapeutic efficacy and reduce the peripheral toxicity of XAV939-L1, we prepared biomimetic brain-targeted XAV939-L1 (XAV939-L1-BT) by coating the XAV939-L1 liposome with neuron–astrocyte hybrid cell membrane (Figure 4A). The resulting XAV939-L1-BT nanoparticle showed an average size of 127.23 ± 7.04 nm in diameter, with a polydispersity index around 0.1, a Zeta potential at approximately −24.52 mV, and a strong exosome signal at 704 nm (Figure 4B–E). To confirm the fusion of the XAV939-L1-exosome with the neuron–astrocyte hybrid membrane, we labeled the neuronal membrane with Cy3.3, the astrocytic membrane with FITC, and the XAV939-L1-exosome with Cy5.5. A super-resolution fluorescent microscope showed triple-labeling of XAV939-L1-BT (Figure 4). To test if XAV939-L1-BT could achieve a high amount of drug accumulation in the brain at a low dosage, we performed in vivo fluorescence imaging via intravenous injection of 2.5 mg/kg XAV939-L1-BT (half dose of XAV939-L1 adopted in Figure 3 to alleviate social dysfunction). As compared with the control (same dose of Cy5.5), XAV939-L1-BT-treated mice showed approximately a 3.4-times increase in Cy5.5 signal in the brain (Figure 4F,G). Meanwhile, a dramatic decrease in the Cy5.5 signal in the liver and a significant decrease in the Cy5.5 signal in the duodenum and jejunum were found in the XAV939-L1-BT-treated mice (Figure 4H–K). These data illustrate the brain-targeted delivery of XAV939-L1-BT by a neuron–astrocyte hybrid cell membrane coating.

### 3.5. Amelioration of Social Deficits in ASD Mice by XAV939-L1-BT with Mitigated Periphery Toxicity

**Figure 5:**
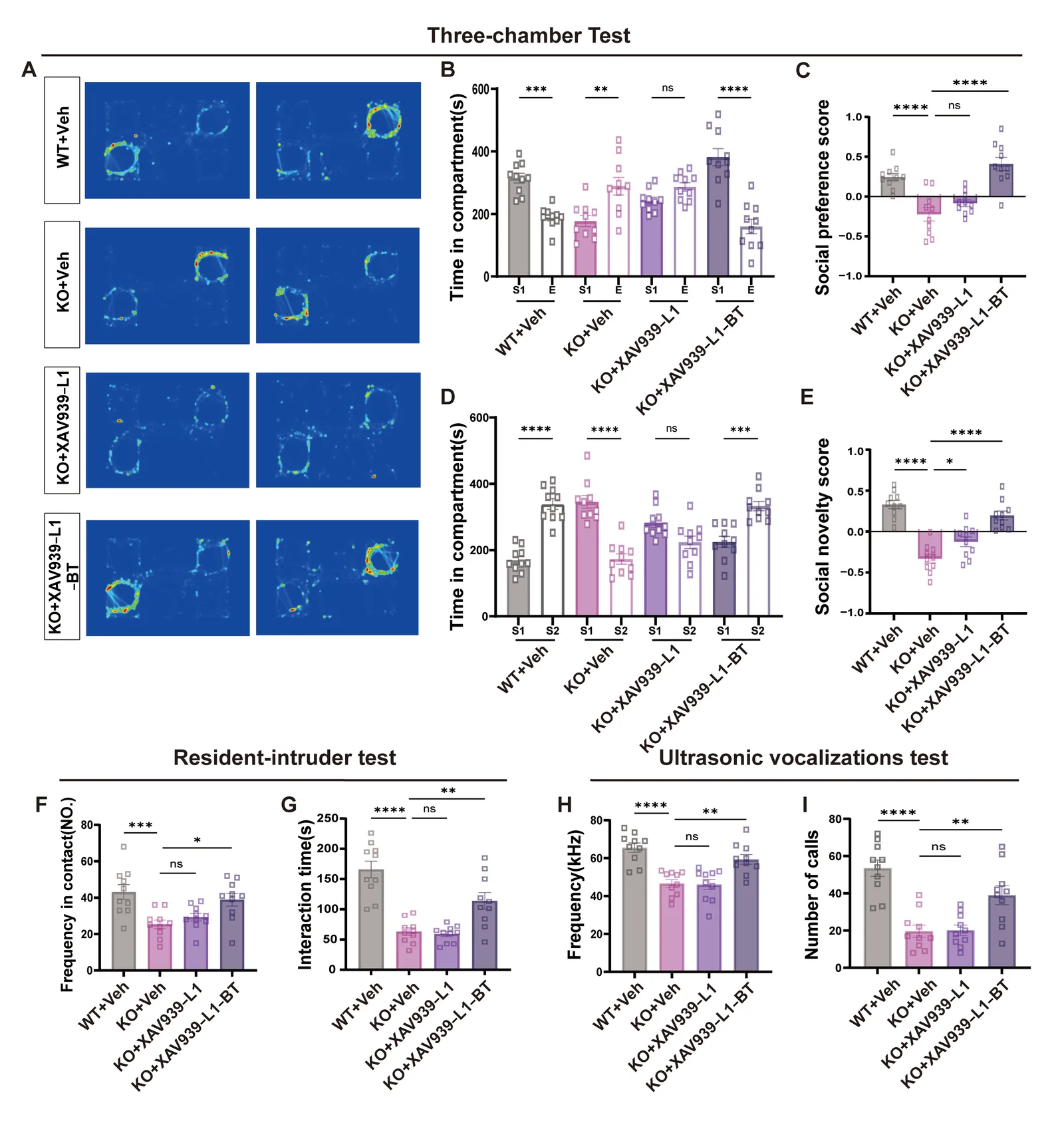
Effects of half-dose XAV939-L1 and XAV939-BT on the social behavior of *Shank3b^-/-^* mice. (**A**–**E**) Three-chamber assay of WT mice treated with vehicle and *Shank3b^-/-^* mice treated with vehicle, XAV939-L1 or XAV939-BT. (**F**,**G**). Resident-intruder assay of WT mice treated with vehicle and *Shank3b^-/-^* mice treated with vehicle, XAV939-L1 or XAV939-BT. (**H**,**I**) Ultrasonic vocalization of WT mice treated with vehicle and *Shank3b^-/-^* mice treated with vehicle, XAV939-L1 or XAV939-BT. Half dose of XAV939-BT significantly improved the social preference, social interaction and social communication of *Shank3b^-/-^* mice. Data are presented as means ± SEM. * *p* < 0.05, ** *p* < 0.01, *** *p* < 0.001, **** *p* < 0.0001. One-way ANOVA followed by Tukey’s post hoc test. n = 10 mice per group. WT, wild type. KO, *Shank3b^-/-^*. Veh, vehicle. ns, non-significance.

Subsequently, we explored the effects of XAV939-L1-BT on the social behavior of *Shank3b^-/-^* mice. Since the above data had demonstrated its efficacy of brain delivery, we evaluated the effects of XAV939-L1-BT (2.5 mg/kg), at half the dose of XAV939-L1 used to alleviate social dysfunction, on the social behavior of *Shank3b^-/-^*. Accordingly, a half dose of XAV939-L1 (2.5 mg/kg) was used as a control. In the three-chamber test, treatment with XAV939-L1-BT significantly enhanced both social preference and social novelty in *Shank3b^-/-^* mice, while treatment with the same dose of uncoated XAV939-L1 had no significant effects on the sociability of *Shank3b^-/-^* mice (Figure 5A–E). In the resident-intruder test, XAV939-L1-BT significantly increased the interaction frequency and interaction time of *Shank3b^-/-^* mice with intruder mice, while the same dose of XAV939-L1 did not influence the social interaction of *Shank3b^-/-^* mice (Figure 5F,G). Considering that the impairment of social communication is a core feature in patients with ASD, we investigated whether XAV939-L1-BT could improve social communication in *Shank3b^-/-^* mice. Ultrasonic vocalization (USV) recordings during interactions between *Shank3b^-/-^* males and wild-type females revealed that XAV939-L1-BT treatment led to a higher number and frequency of USV calls, while the same dose of XAV939-L1 did not (Figure 5H,I). These data demonstrated that XAV939-L1-BT, at a half dose of XAV939-L1, is effective in improving the social function of ASD mice.

**Figure 6:**
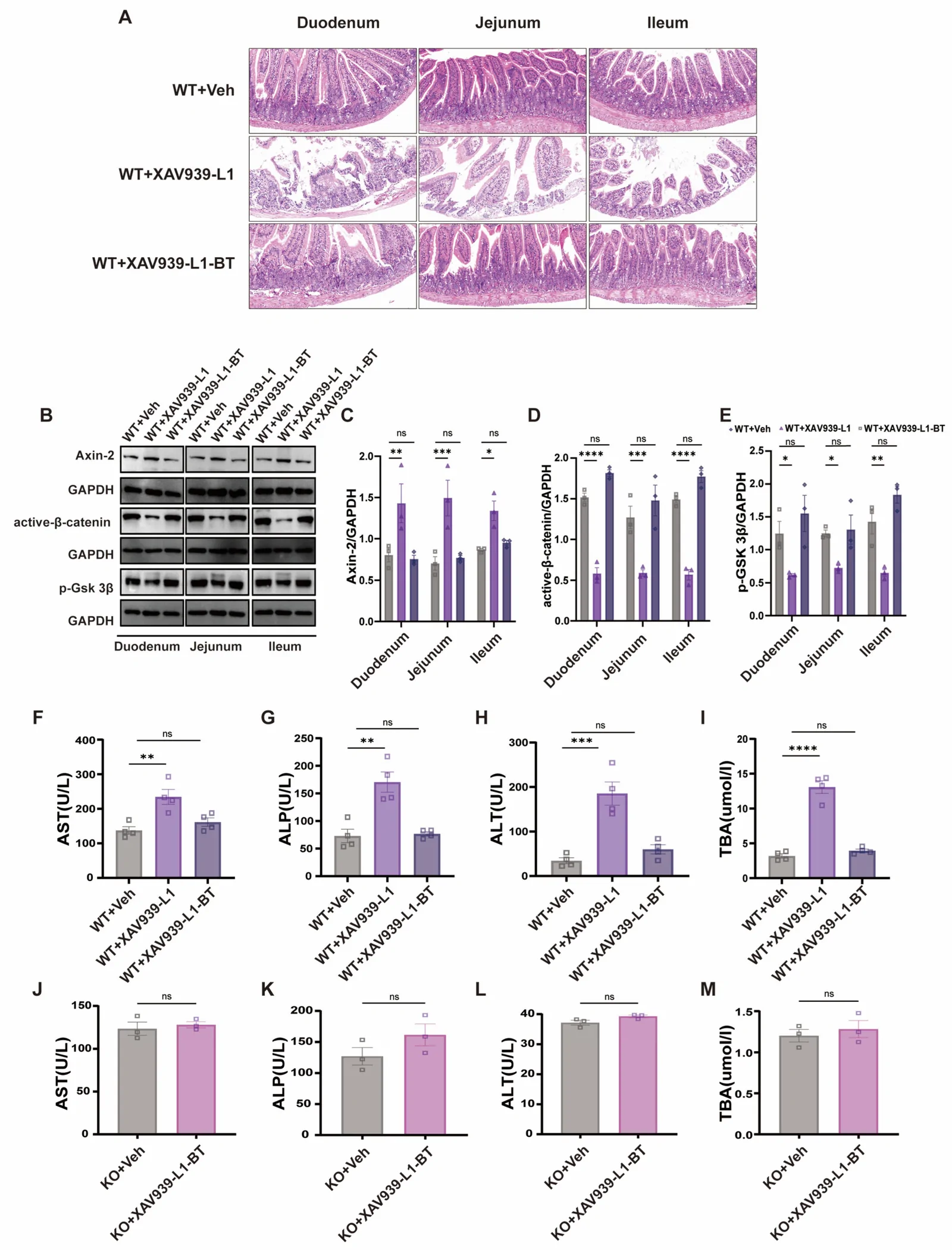
Periphery toxicities of half-dose XAV939-L1 and XAV939-L1-BT. (**A**,**B**) H.E. staining of small intestine following XAV939-L1 or XAV939-L1-BT treatment on WT mice. (**C**–**E**) Western blotting of Axin2, p-GSK-3β and active β-catenin in different segments of small intestine following treatment with XAV939-L1 or XAV939-L1-BT. (**F**–**I**) Serum enzymatic activities of AST, ALP, ALT and TBA in WT mice following 5 days’ treatment with XAV939-L1 or XAV939-L1-BT. (**J**–**M**) Serum enzymatic activities of AST, ALP, ALT and TBA in *Shank3b^-/-^* mice treated with or without XAV939-L1-BT. Half-dose XAV939-L1 still shows hepatic and intestinal toxicities, while XAV939-L1-BT did not. Data are presented as means ± S.E.M. n = 3 mice per group in (**C**–**E**), 4 mice per group in (**F**–**I**), 3 mice per group in (**J**–**M**). * *p* < 0.05, ** *p* < 0.01, *** *p* < 0.001, **** *p* < 0.0001. One-way ANOVA followed by Tukey’s post hoc test. Two-tailed Student’s *t*-test. WT, wild type. KO, *Shank3b^-/-^*. Veh, vehicle. ns, non-signficance.

In the end, we assessed the peripheral toxicity of XAV939-L1-BT and XAV939-L1 at the dose of 2.5 mg/kg in both WT and *Shank3b^-/-^* mice. In WT mice, H.E. staining showed that XAV939-L1 led to an obvious disruption of the intestinal villi at all segments of the intestine, while XAV939 did not (Figure 6A). Western blots showed that XAV939-L1 significantly increased the levels of Axin2 and decreased the levels of active β-catenin and p-GSK-3β in intestinal segments, while the same dose of XAV939-L1-BT did not (Figure 6B–E). In WT mice, XAV939-L1 significantly increased the activities of these enzymes, whereas the same dose of XAV939-L1-BT did not change any of the hepatic enzymes measured (Figure 6F–I). In *Shank3b^-/-^* mice, XAV939-L1-BT exerted no significant effects on any of the enzymes examined (Figure 6J–M). These data demonstrate that the XAV939-L1-BT formulation induces minimal peripheral toxicity at a dose that is beneficial for social function.

## 4. Discussion

In the present study, we synthesized three XAV-939 derivatives with lipid modification. After screening their effectiveness in inhibiting Wnt/glycolysis in both 293T cells and primary *Shank3b^-/-^* neurons, we identified an alkyl ester prodrug of XAV939, named XAV939-L1, as the most potent dual inhibitor of Wnt/glycolysis. Compared to XAV939, XAV939-L1, by itself, can alleviate social dysfunction in ASD mice by intravenous injection, suggesting the enhancement of bioavailability. To reduce the periphery side-effects, we prepared biomimetic brain-targeting XAV939-L1 by coating the compound with a neuron–astrocyte hybrid cell membrane, and achieved higher brain enrichment, better functional improvement and fewer side effects.

XAV939 was originally identified as an antitumor compound [22,23] and, later, proposed as a powerful agent for promoting remyelination [24]. Owing to its poor lipophilicity and insufficient transmembrane permeability, XAV939 can hardly penetrate the blood–brain barrier (BBB) to reach therapeutic concentration in the brain. In addition, the unfavorable lipophilic–hydrophilic profile of XAV939 results in low oral absorption efficiency and rapid metabolism following vein delivery. Although local injection of XAV939 exhibits significant social improvement effects [16], these shortcomings severely restrict its clinical translation.

To address these limitations, we proposed a rational lipid-based prodrug strategy [25], which involves the covalent conjugation of a lipophilic moiety to a polar functional group on the scaffold of XAV939. This modification can temporarily mask the polar group, thereby optimizing the overall lipophilicity and the oil–water partition coefficient of the molecule. The enhanced lipophilicity is expected to facilitate passive transcellular diffusion across the BBB. Based on this hypothesis, we performed lipophilic prodrug structural modification of XAV939 via esterification, aiming to simultaneously overcome the druggability barriers of low bioavailability and poor BBB penetration. Both our in vitro and in vivo data supported the effectiveness of this strategy, indicating XAV939-L1 as a potential prodrug for further investigation. Considering the broad range of phenotypes of ASD, it is possible that XAV939-L1 may have other benefits beyond improving social behavior, which is to be investigated in the future.

Inhibiting Wnt signaling and glycolysis for treating ASD is relatively “safe” for brain function, because Wnt/glycolysis levels are low in mature neurons [26,27]. Our previous study has demonstrated that Wnt/glycolysis signaling declines dramatically to a basal level at postnatal 2–3 weeks in the cerebral cortex [16]. Therefore, the potential side effects in peripheral tissues should be considered for the drug development of XAV939-L1. The observed social improvement is not the off-target effects of XAV939-L1, since XAV939-L1 treatment did not significantly influence the animal’s basal movement and motor–sensory coordination, although *Shank3b^-/-^* mice showed a certain motor deficiency due to the defects in the cerebellum and neuromuscular junction [28,29]. To reduce the peripheral side effects, we adopted a hybrid cell membrane-coating strategy to enhance brain delivery. In past decades, many methods have been developed for delivering drugs through the BBB [30,31]. Most of them are chemical-based, with limited drug loading and poor biocompatibility. Recently, cell membrane-coating has been adopted for brain targeting, which is easy to penetrate the BBB and is taken up by neural cells [30,32,33]. In addition, cell membrane coating is advantageous in larger drug loading, longer drug circulation, and less immune response [34]. Almost all neural cells, such as microglia, astrocytes, oligodendrocyte progenitor cells, and neurons, have been tested as coating material to make nanoparticles, depending on the pathological conditions and the target cells to deliver the drug [35]. The neural cell membrane-coated nanoparticles do not activate the microglia and are taken up preferably by their source cells. To penetrate the BBB, astrocyte coating is often preferred because astrocytes are the major component of the BBB [36]. Considering that Wnt signaling and glycolysis are mainly activated in neuronal cells in ASD models [16,37], we adopted the neuron–astrocyte hybrid cell membrane-coating strategy, which is advantageous in ensuring both efficient BBB penetration and neuronal accumulation of the compound. The high intensity of drug fluorescence in the brain, effectiveness in improving social function and dramatic reduction of peripheral toxicity demonstrated the success of this strategy in an animal study. The shortcomings of this strategy for clinical application are the source of the neuronal and astrocytic membranes and the chronic immune response if allogeneic neuron–astrocyte membranes were to be repeatedly used. In the future, it is worthy to test the neuron–astrocyte membranes obtained from patient-derived induced pluripotent stem cells, which can overcome these shortcomings, or other biomimetic materials. The effectiveness–dosage relationship and the effects of XAV939-L1 on human neurons are also to be investigated. Overall, our data suggested that an esterified XAV939 and its brain-targeted formula could be a potential prodrug for treating ASD-associated social dysfunction.

## Data Availability

The original contributions presented in this study are included in the article/Supplementary Material. Further inquiries can be directed to the corresponding authors.

## Supplementary Materials

The following supporting information can be downloaded at https://www.mdpi.com/article/10.3390/pharmaceutics18080951/s1: Figure S1: ^1^H NMR, ^13^C NMR and HRMS spectra of XAV939, XAV939-L1, XAV939-L2 and XAV939-L3. Figure S2: HPLC analysis of XAV939-L1 degradation in brain tissue. Figure S3: Effects of XAV939-L1 on the basal activity of WT and *Shank3b^-/-^* mice. Figure S4. Representative super resolution fluorescence microscopic images of XAV939-L1-BT

## Abbreviations

The following abbreviations are used in this manuscript:

ALP	Alkaline phosphatase
ALT	Alanine aminotransferase
ASD	Autism spectrum disorder
AST	Activities of aspartate aminotransferase
BBB	Blood–brain barrier
ECAR	extracellular acidification rate
ECL	Enhanced chemiluminescence
ESI	Electrospray ionization
H.E.	Hematoxylin and eosin
HRMS	High-resolution mass spectra
PDI	Polydispersity index
SPC	Soybean phosphatidylcholine
SEM	Standard error of the mean
TBA	Total bile acid
TEM	Transmission electron microscope
TLC	Thin-layer chromatography
TMS	Tetramethylsilane
USV	Ultrasonic vocalization
