# Supplementary material for: Biomimetic Brain-Targeted Delivery of Esterified XAV939 for Treating Autism-Associated Social Deficits
Source: Pharmaceutics. 2026 Jul 31;18(8):951. doi: 10.3390/pharmaceutics18080951 (PMC13516923; doi:10.3390/pharmaceutics18080951)
Supplement: Supplementary file 1 [file pharmaceutics-18-00951-s001.zip › pharmaceutics-4306504-supplementary.pdf]

Biomimetic Brain-Targeted Delivery of Esterified XAV939 for Treating Autism-Associated Social Deficits

Hanze Liu, Ya-Rong Wang, Mengmeng Wang, Tiantian Yu, Daozhou Liu, Jing Wang, Yang Gao, Yuanyiyang Hu, Hongyu Ma, Feng Gao, Shengxi Wu, Zhao Wei and Yazhou Wang

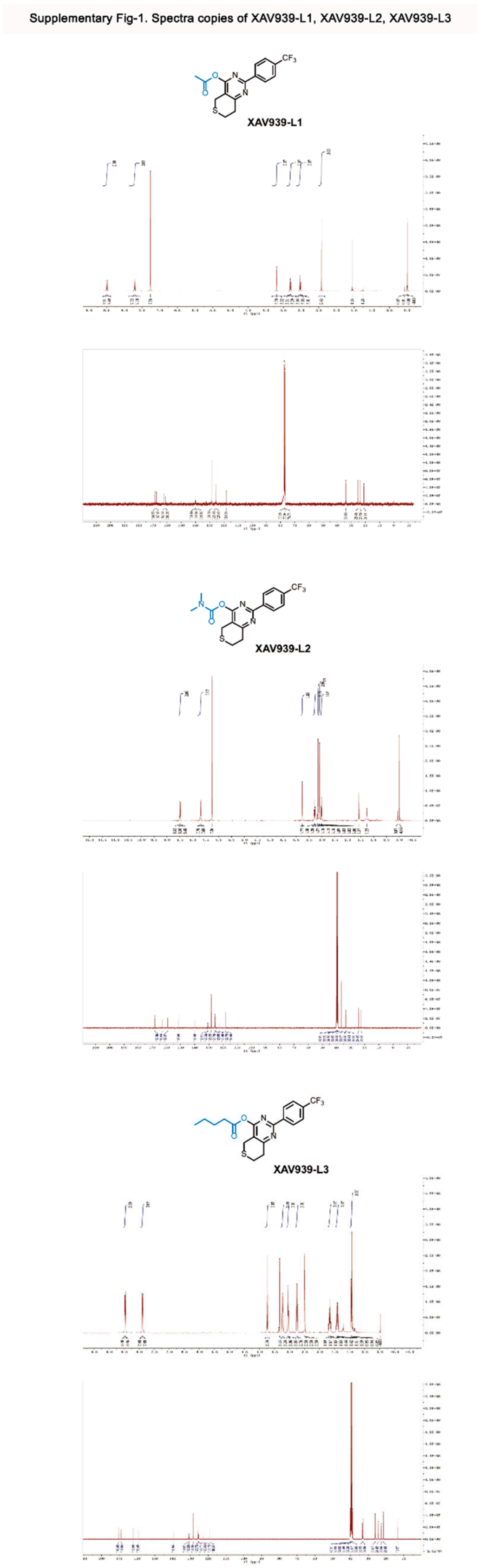

Figure S1. ^{1}H NMR, ^{13}C NMR and HRMS spectra of XAV939, XAV939-L1, XAV939-L2 and XAV939-L3.

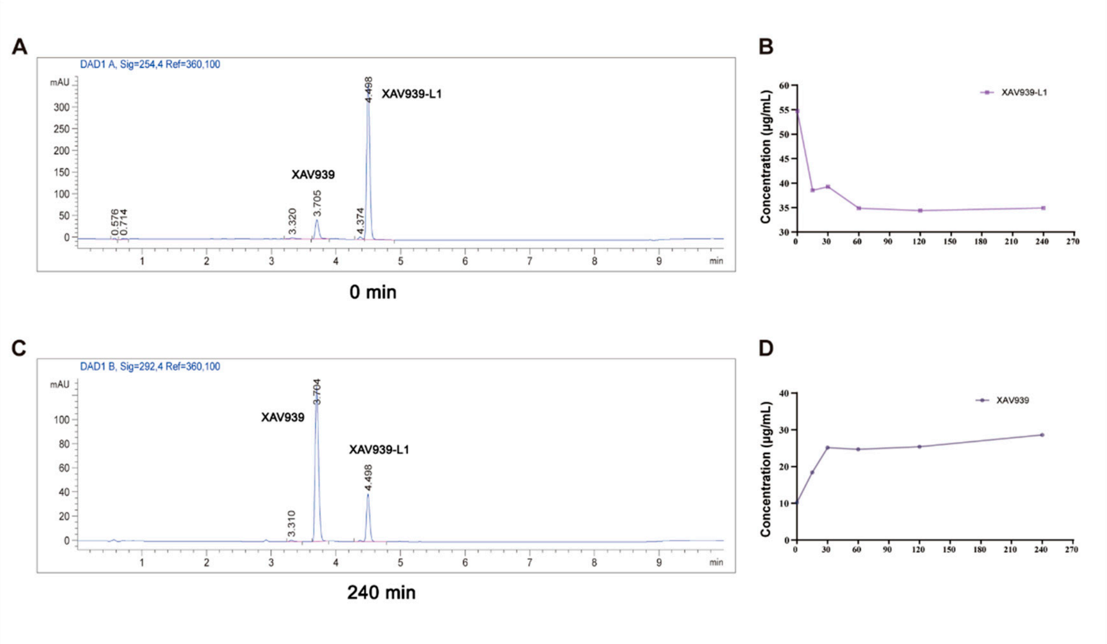

Figure S2. HPLC analysis of XAV939-L1 degradation in brain tissue. (**A**, **C**) Typical traces of XAV939 and XAV939-L1 at 0 min and 240 min of XAV939-L1-brain tissue incubation. (**B**, **D**) Concentrations of XAV939-L1 and XAV939 at different time points of XAV939-L1-brain tissue incubation.

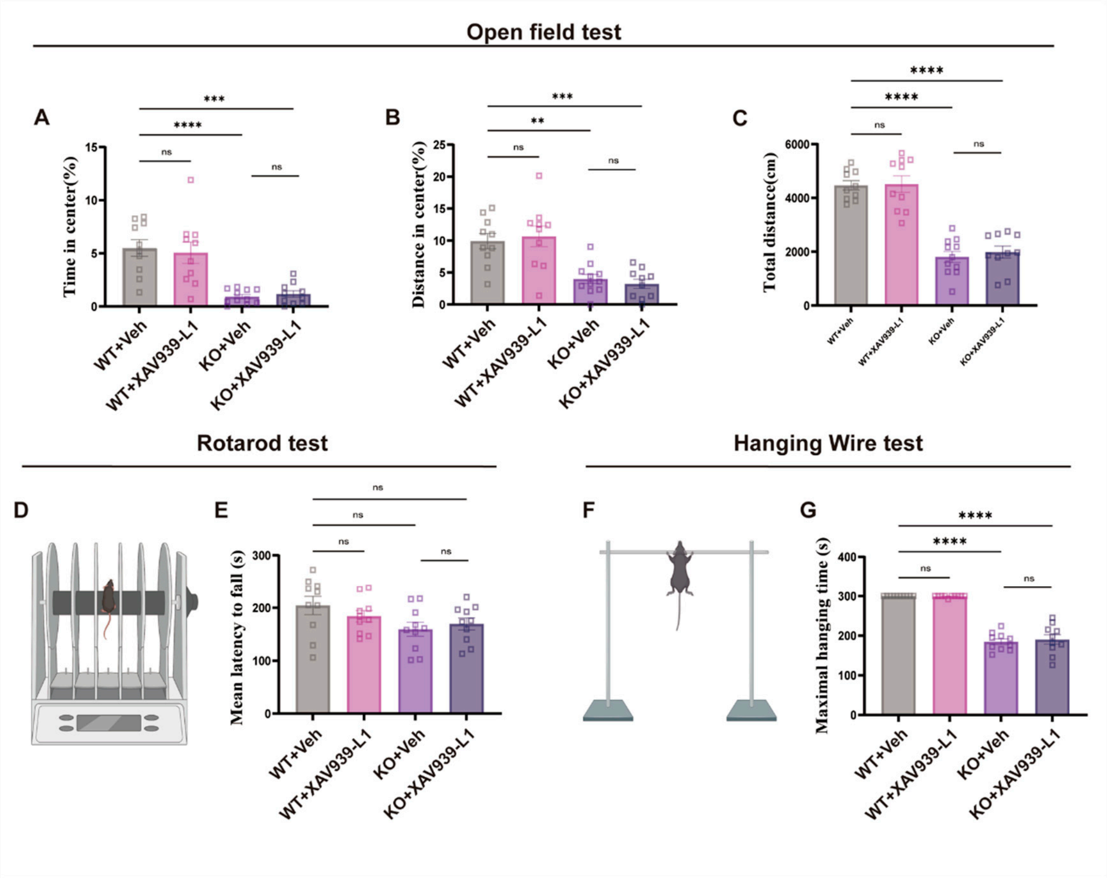

Figure S3. Effects of XAV939-L1 on the basal activity of WT and *Shank3b*$^{-/-}$ mice. (**A** – **C**) Open-field test. (**D,E**) Rotarod test. Created in BioRender. Liu, H. (2026) https://BioRender.com/u0c3i4e (**F,G**) Hanging wire test. Created in BioRender. Liu, H. (2026) https://BioRender.com/pifjdgb

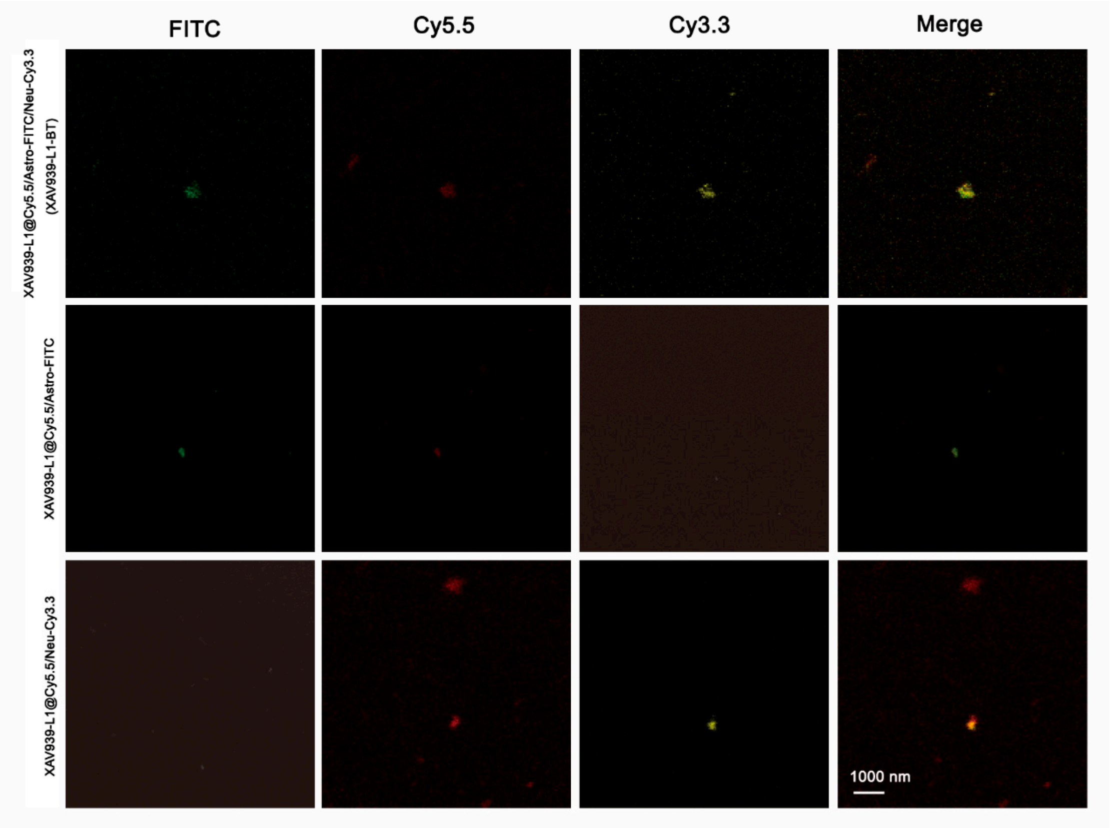

Figure S4. Representative super resolution fluorescence microscopic images of XAV939-L1-BT which composed XAV939-L1-exosomes labeled by Cy5.5, neuronal membrane labeled by Cy3.3 and astrocytic membrane labeled by FITC.
